# Supplementary material for: Metrics for describing dyadic movement: a review
Source: Mov Ecol. 2018 Dec 27;6:26. doi: 10.1186/s40462-018-0144-2 (PMC6307229; doi:10.1186/s40462-018-0144-2)
Supplement: Supplementary file 1 — Graphical examples of two kernel functions for Proximity metrics. (PDF 89 kb) [file 40462_2018_144_MOESM1_ESM.pdf]

Additional file 1: Graphical examples of two kernel functions for Proximity metrics

Rocio Joo

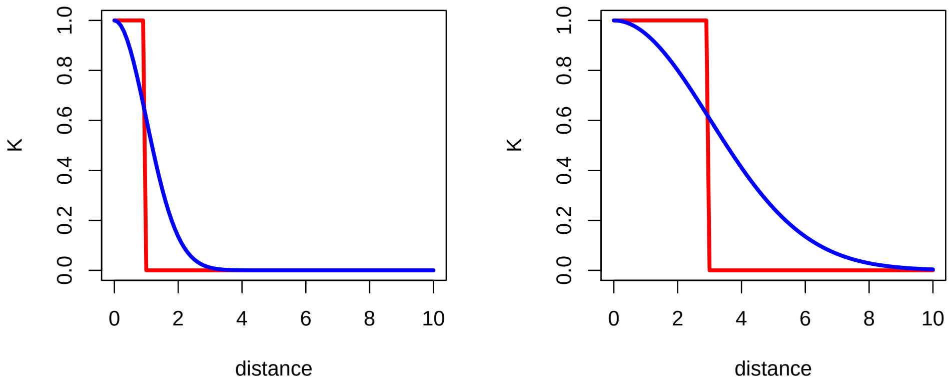

Figure 1: Illustration of differences between to kernel values with the same δ value. The red and blue solid lines corresponds to $K_\delta(x, y) = \mathbb{1}_{\{\|x-y\|<\delta\}}$ and $K_\delta(x, y) = \exp\left(-\|x-y\|^2/(2\delta^2)\right)$, respectively. Left panel: $\delta = 1$. Right panel: $\delta = 3$.
