# Supplementary material for: Metrics for describing dyadic movement: a review
Source: Mov Ecol. 2018 Dec 27;6:26. doi: 10.1186/s40462-018-0144-2 (PMC6307229; doi:10.1186/s40462-018-0144-2)
Supplement: Supplementary file 2 — Cs1 requirements to take large negative values. (PDF 359 kb) [file 40462_2018_144_MOESM2_ESM.pdf]

Additional file 2: Cs1 requirements to take large negative values

Rocio Joo

The Coefficient of Sociality (Cs) compares the mean distance between simultaneous pairs of fixes (D_O) against the mean distance between all permutations of all fixes (D_E).

$$Cs = \frac{D_E - D_O}{D_E + D_O} = 1 - 2\frac{D_O}{D_E + D_O}, \tag{1}$$

where

$$D_O = \Big(\sum_{t=1}^{T} d_t^{A,B} \Big) / T,$$

and

$$D_E = \Big(\sum_{t_1=1}^{T} \sum_{t_2=1}^{T} d_{t_1,t_2}^{A,B} \Big) / T^2.$$

Let d_{ij} be the distance between the locations of A at time i and B at time j. Then, D_O and D_E can be expressed as in equations 2 and 4.

$$D_O = \sum_{i_1=1}^{T} d_{ii} / T \tag{2}$$

$$D_{\bar{O}} = \sum_{\substack{i,j \in [1,T] \\ i \neq j}} d_{ij} / (T^2 - T) \tag{3}$$

$$D_E = \frac{D_O}{T} + \frac{(T-1)}{T} D_{\bar{O}} \tag{4}$$

where $D_{\bar{O}}$ is defined in equation 3 and corresponds to the average distance between the exclusively permuted points without taking into account the simultaneous fixes. Using those equations, we can replace D_O and D_E in equation 1 when $Cs1 = -\alpha$ ($\alpha > 0$) and obtain:

$$\frac{D_{\bar{O}}}{D_O} = \frac{T(1-\alpha)}{(T-1)(1+\alpha)} - \frac{1}{T-1} \tag{5}$$

It means that, for instance, for $Cs1 = -0.5$ and when T is large, $D_{\bar{O}}$ would have to be approximately a third of D_O, thus a third of the average distance computed only at simultaneous fixes. Fig. 1 shows the values of $D_{\bar{O}}/D_O$ ratios needed to attain the whole range of Cs negative values. Most of those scenarios are very unlikely.

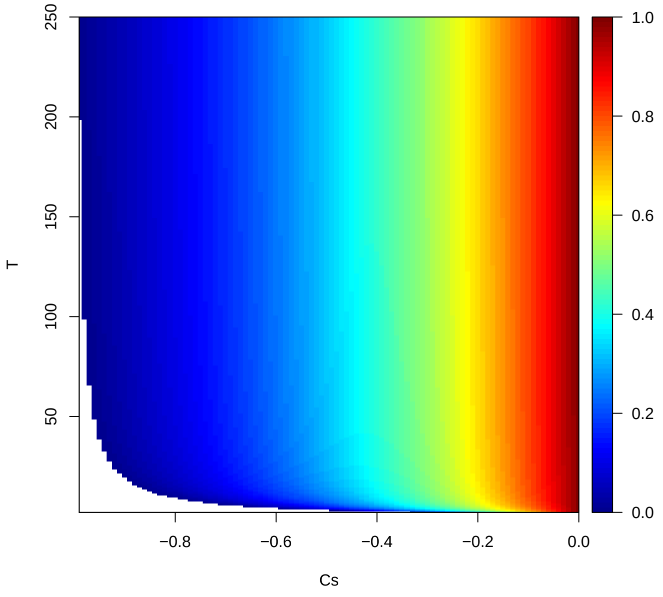

Figure 1: Computed ratios $D_{\bar{O}}/D_O$ needed for obtaining the Cs1 negative values (x-axis, from -0.99 to 0) for each series length T (y-axis, from 2 to 250). The blank spaces correspond to infeasible situations.
