# Supplementary material for: Metrics for describing dyadic movement: a review
Source: Mov Ecol. 2018 Dec 27;6:26. doi: 10.1186/s40462-018-0144-2 (PMC6307229; doi:10.1186/s40462-018-0144-2)
Supplement: Supplementary file 3 — Lixn: Table for computing probabilities. (PDF 119 kb) [file 40462_2018_144_MOESM3_ESM.pdf]

Additional file 3: Lixn: Table for computing probabilities

Rocio Joo

Table 1: Probabilities of finding individuals A and B simultaneously or not in the reference areas.

Probability	With individual areas	With expected frequency
p_{AB}	$\frac{S_{AB}}{S_A} \times \frac{S_{AB}}{S_B}$	$\frac{n_A}{T} \times \frac{n_B}{T}$
p_{A0}	$\frac{S_{AB}}{S_A} \times \left(1 - \frac{S_{AB}}{S_B}\right)$	$\frac{n_A}{T} \times \left(1 - \frac{n_B}{T}\right)$
p_{0B}	$\left(1 - \frac{S_{AB}}{S_A}\right) \times \frac{S_{AB}}{S_B}$	$\left(1 - \frac{n_A}{T}\right) \times \frac{n_B}{T}$
p_{00}	$\left(1 - \frac{S_{AB}}{S_A}\right) \times \left(1 - \frac{S_{AB}}{S_B}\right)$	$\left(1 - \frac{n_A}{T}\right) \times \left(1 - \frac{n_B}{T}\right)$

Note: p_{AB} is the probability of finding A and B simultaneously in the reference area S_{AB} (when a subscript is 0, it represents the absence of the corresponding individual from the reference area); S_A and S_B are the individual areas of A and B, respectively, however they are defined; n_A and n_B are the number of observed fixes of A and B (respectively) in the reference area; T is the number of fixes of each individual.
