# Supplementary material for: Metrics for describing dyadic movement: a review
Source: Mov Ecol. 2018 Dec 27;6:26. doi: 10.1186/s40462-018-0144-2 (PMC6307229; doi:10.1186/s40462-018-0144-2)
Supplement: Supplementary file 4 — How to define the ellipse of the potential path area. (PDF 62 kb) [file 40462_2018_144_MOESM4_ESM.pdf]

Additional file 4: How to define the ellipse of the potential path area

Rocio Joo

At each step, and for each individual, an ellipse is drawn; two consecutive records are used as focal points and the length of the major axis of the ellipse is computed as $D = \phi \times \Delta t$ (ϕ is the maximum velocity of the individual and Δt is the time difference between the consecutive records). The area of the ellipse represents the potential path area [1].

References

[1] Long, J. A. and Nelson, T. A. (2012). Time geography and wildlife home range delineation. Journal of Wildlife Management, 76(2):407–413.
