# Supplementary material for: Metrics for describing dyadic movement: a review
Source: Mov Ecol. 2018 Dec 27;6:26. doi: 10.1186/s40462-018-0144-2 (PMC6307229; doi:10.1186/s40462-018-0144-2)
Supplement: Supplementary file 5 — Metrics derived for each case scenario. (PDF 63 kb) [file 40462_2018_144_MOESM5_ESM.pdf]

Additional file 5: Metrics derived for each case scenario

Rocio Joo

Table 1: First subset of metrics

	$Prox_1$	$Prox_2$	$Prox_3$	Cs	HAI_1	HAI_2	HAI_3	$L_{ixn}T$	jPPA	$CSEM_1$	$CSEM_2$	$CSEM_3$	r_{Lon}	r_{Lat}	r_{Lonlat}	r_{Speed}	DI_d	DI_θ	DI
1	1.00	1.00	1.00	0.77	0.89	0.89	0.89	0.92	0.93	1.00	1.00	1.00	1.00	1.00	1.00	1.00	1.00	1.00	1.00
2	0.70	1.00	1.00	0.61	1.00	1.00	1.00	1.00	0.86	0.56	1.00	1.00	1.00	1.00	1.00	1.00	0.82	1.00	0.82
3	0.40	0.80	1.00	0.44	1.00	1.00	1.00	1.00	0.87	0.22	0.44	1.00	-1.00	1.00	-0.00	1.00	1.00	0.57	0.57
4	0.60	0.80	1.00	0.45	0.57	0.73	0.73	0.71	0.88	0.33	0.67	1.00	-0.15	0.90	0.38	0.03	0.73	0.44	0.36
5	0.60	0.80	1.00	-0.15	1.00	1.00	1.00	1.00	0.90	0.44	0.56	1.00	-1.00	-1.00	-1.00	1.00	1.00	-1.00	-1.00
6	0.60	0.90	1.00	-0.12	0.67	0.67	0.67	0.81	0.80	0.44	0.56	1.00	-0.93	-0.99	-0.96	1.00	0.59	-1.00	-0.59
7	0.00	0.00	1.00	0.21					0.67	0.00	0.00	1.00	1.00	1.00	1.00	1.00	1.00	1.00	1.00
8	0.00	0.00	0.80	0.18					0.59	0.00	0.00	0.56	1.00	1.00	1.00	1.00	0.82	1.00	0.82
9	0.60	0.60	0.80	0.35	1.00	1.00	1.00	1.00	0.68	0.56	0.56	0.78	-1.00	1.00	0.00	1.00	1.00	0.79	0.79
10	0.50	0.50	0.80	0.34	0.91	0.91	0.91	0.92	0.62	0.44	0.44	0.78	-0.77	0.98	0.10	0.39	0.78	0.71	0.55
11	0.40	0.60	0.60	-0.05	0.89	0.92	0.92	0.92	0.60	0.22	0.56	0.56	-1.00	-1.00	-1.00	1.00	1.00	-1.00	-1.00
12	0.50	0.60	0.60	-0.03	1.00	1.00	1.00	1.00	0.64	0.44	0.56	0.56	-1.00	-1.00	-1.00	1.00	0.82	-1.00	-0.82
13	0.00	0.00	0.00	0.01					0.26	0.00	0.00	0.00	1.00	1.00	1.00	1.00	1.00	1.00	1.00
14	0.00	0.00	0.00	0.09					0.42	0.00	0.00	0.00	1.00	1.00	1.00	1.00	0.82	1.00	0.82
15	0.00	0.00	0.00	0.05					0.29	0.00	0.00	0.00	-1.00	1.00	0.00	1.00	1.00	0.79	0.79
16	0.00	0.00	0.20	0.12					0.45	0.00	0.00	0.00	-0.15	0.90	0.38	0.03	0.73	0.44	0.36
17	0.00	0.20	0.60	-0.03					0.41	0.00	0.00	0.56	-1.00	-1.00	-1.00	1.00	1.00	-1.00	-1.00
18	0.00	0.10	0.60	-0.03					0.44	0.00	0.00	0.56	-1.00	-1.00	-1.00	1.00	0.82	-1.00	-0.82
