# Supplementary material for: Metrics for describing dyadic movement: a review
Source: Mov Ecol. 2018 Dec 27;6:26. doi: 10.1186/s40462-018-0144-2 (PMC6307229; doi:10.1186/s40462-018-0144-2)
Supplement: Supplementary file 6 — Summary figures for proximity-speed and proximity-coordination scenarios. (PDF 59 kb) [file 40462_2018_144_MOESM6_ESM.pdf]

Additional file 6: Summary figures for proximity-speed and proximity-coordination scenarios

Rocio Joo

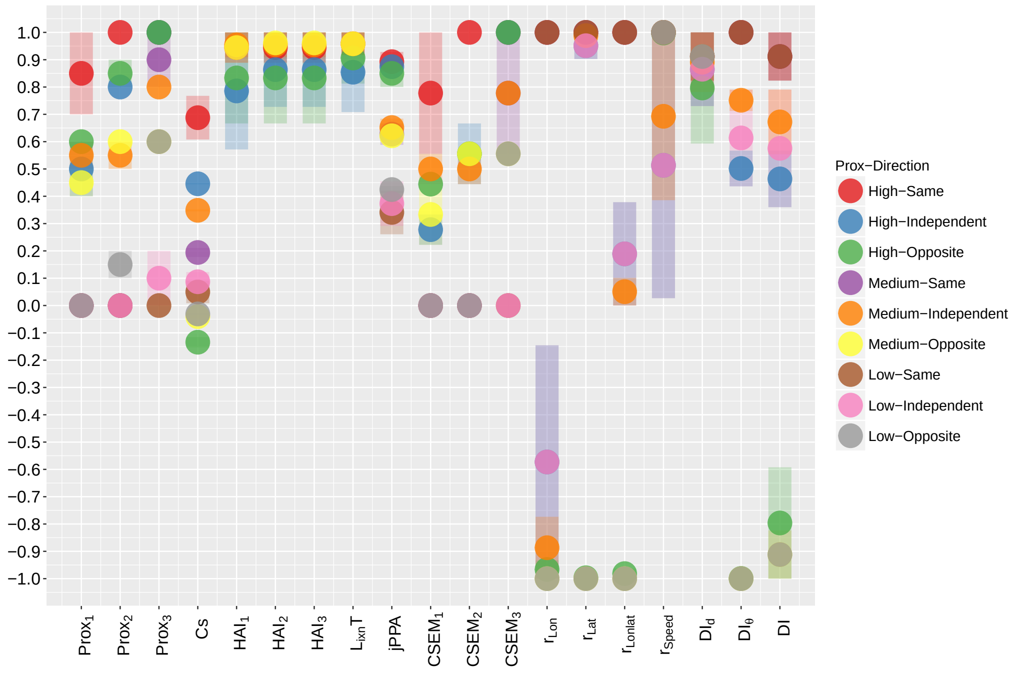

Figure 1: Median (solid circle), minimum and maximum (bar) for each metric by combined category of proximity and direction coordination.

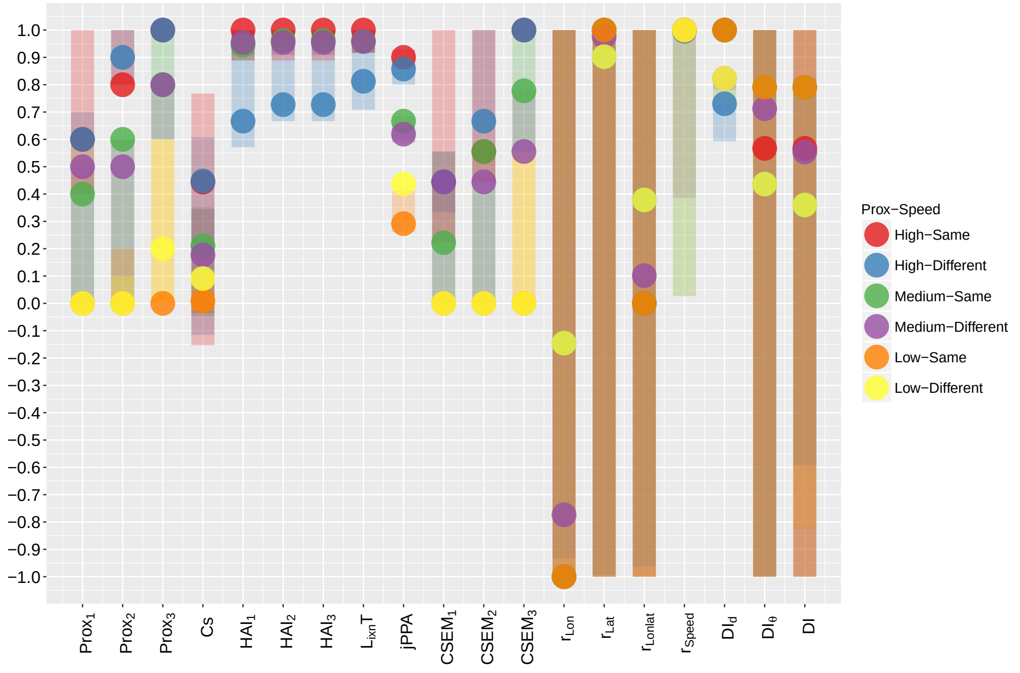

Figure 2: Median (solid circle), minimum and maximum (bar) for each metric by combined category of proximity and speed coordination.
