# Supplementary material for: Metrics for describing dyadic movement: a review
Source: Mov Ecol. 2018 Dec 27;6:26. doi: 10.1186/s40462-018-0144-2 (PMC6307229; doi:10.1186/s40462-018-0144-2)
Supplement: Supplementary file 7 — Computational cost of each metric. (PDF 91 kb) [file 40462_2018_144_MOESM7_ESM.pdf]

Additional file 7: Computational cost of each metric

Rocio Joo

1 Simulation Procedure

We simulated 50000 dyads, with trajectories following a Brownian motion. Each trajectory was composed of N fixes. We measured the total CPU time spend in the computation of each metric for the 50000 dyads. This analysis was performed for $N = \{100, 500, 1000, 5000, 10^4, 10^5\}$, and it was repeated 5 times (for each N). Because individuals were given freedom to go in any direction, no assumption on a reference area was made, thus $L_{ixn}T$ and HAI were not considered in this part of the analysis.

2 Code

The R code to compute the CPU time is in the function SimulationTempsCalcul in https://github.com/rociojoo/MetricsDyadJM/. The calculation was parallelized using 4 cores (Processor: Intel Core i7-8550U CPU 1.80GHz × 8).

3 Results

Table 1: Mean CPU time for computing each metric for 50000 simulated dyads (in seconds); the minimum and maximum values are in brackets.

Metric	Number of fixes					
	100	500	1000	5000	10000	100000
$Prox$	0.07	0.07	0.05	0.06	0.05	0.05
	$[0.07, 0.07]$	$[0.07, 0.07]$	$[0.04, 0.06]$	$[0.06, 0.07]$	$[0.05, 0.06]$	$[0.05, 0.06]$
Cs	0.52	0.50	0.57	0.56	0.53	0.54
	$0.50, 0.54]$	$[0.50, 0.51]$	$[0.52, 0.66]$	$[0.51, 0.60]$	$[0.51, 0.55]$	$[0.52, 0.56]$
$jPPA$	67.9	68.9	161	162	156	164
	$[64.4, 69.0]$	$[68.6, 69.5]$	$[147, 168]$	$[153, 170]$	$[152, 163]$	$[158, 169]$
r_{Lonlat}	0.10	0.10	0.09	0.10	0.09	0.10
	$[0.10, 0.10]$	$[0.10, 0.11]$	$[0.08, 0.10]$	$[0.09, 0.11]$	$[0.09, 0.10]$	$[0.09, 0.11]$
r_{Speed}	0.13	0.13	0.12	0.13	0.12	0.14
	$[0.13, 0.13]$	$[0.13, 0.13]$	$[0.11, 0.13]$	$[0.12, 0.13]$	$[0.12, 0.13]$	$[0.12, 0.16]$
$CSEM$	0.47	0.47	0.94	0.93	0.89	0.94
	$[0.47, 0.47]$	$[0.47, 0.47]$	$[0.85, 1.00]$	$[0.88, 0.97]$	$[0.87, 0.92]$	$[0.87, 1.03]$
DI	0.17	0.17	0.17	0.18	0.17	0.19
	$[0.17, 0.18]$	$[0.16, 0.17]$	$[0.16, 0.19]$	$[0.17, 0.20]$	$[0.17, 0.19]$	$[0.17, 0.21]$
DI_d	0.13	0.13	0.12	0.13	0.12	0.13
	$[0.13, 0.13]$	$[0.13, 0.13]$	$[0.10, 0.14]$	$[0.11, 0.18]$	$[0.11, 0.15]$	$[0.12, 0.14]$
DI_θ	0.15	0.15	0.16	0.15	0.14	0.15
	$[0.15, 0.15]$	$[0.14, 0.15]$	$[0.13, 0.21]$	$[0.14, 0.19]$	$[0.14, 0.16]$	$[0.14, 0.16]$
