# Supplementary material for: Metrics for describing dyadic movement: a review
Source: Mov Ecol. 2018 Dec 27;6:26. doi: 10.1186/s40462-018-0144-2 (PMC6307229; doi:10.1186/s40462-018-0144-2)
Supplement: Supplementary file 8 — Principal component analysis of the metrics for the case scenarios. (PDF 190 kb) [file 40462_2018_144_MOESM8_ESM.pdf]

Additional file 8: Principal component analysis of the metrics for the case scenarios

Rocio Joo

The initial data table for the PCA is the one in Additional file 5. We discarded HAI and $L_{ixn}T$ because of the missing values. We keep only one of the Prox and CSEM metrics ($Prox_3$ and $CSEM_3$), and kept r_{Lonlat} but not r_{Lat} nor r_{Lon} for the PCA. The final data table was thus composed of 9 variables and 18 individuals.

PCA was performed using the FactoMineR package [1]. We retained 3 components since they explained 90.1% of the total variance. The loadings of each metric regarding each component are detailed in Table 1 and represented in Figure 1. The first component (38.8% of the variance) was highly correlated to metrics associated to coordination in direction. The second component (34.6% of the variance) was highly correlated to proximity-related metrics. The third component (16.7% of the variance) was highly correlated to the metrics associated to coordination in speed.

References

[1] Husson, F., Josse, J., Le, S., and Mazet, J. (2013). FactoMineR: multivariate exploratory data analysis and data mining with R. R package version 1.25.

Table 1: Metric loadings for the three principal components

	PC1	PC2	PC3
$Prox_3$	0.13	0.97	0.05
Cs	0.85	0.36	0.01
jPPA	0.24	0.93	-0.02
$CSEM_3$	0.13	0.97	0.11
r_{Lonlat}	0.92	-0.26	-0.00
r_{Speed}	-0.21	0.00	0.86
DI_d	0.08	-0.13	0.87
DI_θ	0.95	-0.29	0.04
DI	0.94	-0.27	0.05

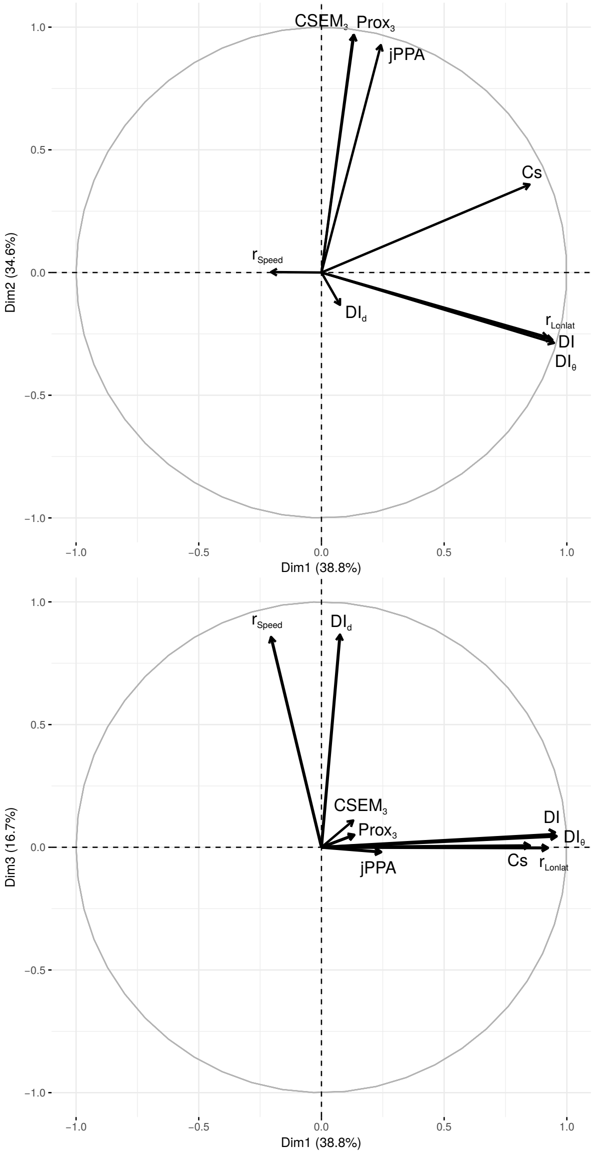

Figure 1: Metric projection in the principal component planes.
